# Supplementary material for: Are carbon emissions trading and green financial instruments synergistic? -Comprehensive quantitative research based on content analysis
Source: PLoS One. 2024 Mar 7;19(3):e0298601. doi: 10.1371/journal.pone.0298601 (PMC10919601; doi:10.1371/journal.pone.0298601)
Supplement: S1 Data — (ZIP) [file pone.0298601.s001.zip › Data/地级市Z_F工作报告.zip!/╣π╢1⁄2/╔ε█┌╩╨/└┤╫╘╣1⁄2╓┌║┼í╛┬φ┐╦╩2╛▌═°í┐_2005.docx]

各位代表：

　　现在，我受市长李鸿忠同志委托，代表市人民政府向大会作政府工作报告，请予审议，并请各位政协委员和其他列席人员提出意见。

　　一、过去五年工作回顾

　　本届政府任期的五年，是深圳进入新世纪的头五年，也是深圳发展历程中很不平凡的五年。我们在党中央、国务院、省委、省政府和市委的正确领导下，以邓小平理论和“三个代表”重要思想为指导，按照中央和省对深圳发展的新要求，认真贯彻落实科学发展观，统一思想，坚定信心，振奋精神，扎实工作，战胜种种困难和挑战，实现了经济快速健康发展和社会全面进步，圆满完成了市三届人大一次会议确定的目标任务。

　　（一）综合实力不断增强，经济发展再上新台阶。五年来，本市生产总值翻了一番多，连续实现了2000、3000亿元的跨越，2004年达到3423亿元，提前两年实现市三届人大一次会议确定的发展目标。人均生产总值突破7000美元。工业增加值增长1.6倍。外贸进出口总额增长近两倍，出口总额达778亿美元，继续保持全国大中城市首位。五年累计完成固定资产投资4100多亿元，超过此前20年的总和，建成一批事关全局和长远发展的基础设施项目，发展后劲大大增强。累计向中央财政贡献2700多亿元，相当于前20年的3倍。

　　高新技术、现代物流、金融三大支柱产业规模和实力进一步壮大。高新技术产品产值增长近3倍，达到3267亿元，占规模以上工业总产值50.2%。高新技术产品出口351亿美元，占全市出口总额45%，占全国高新技术产品出口1/5强。港口集装箱吞吐量1366万标箱，在世界集装箱枢纽港的排名由1999年的第十一位跃升至第四位。机场旅客和货邮行吞吐量分别进入全球100强和50强，初步确立了区域性航空运输中心地位。物流业发展迅速，现代物流配送体系初步形成。金融机构本外币存、贷款余额分别达到8115亿元和6547亿元。深圳证券交易所获准设立中小企业板。保险、基金、创投业健康发展，在全国占有重要地位。

　　（二）落实科学发展观，走上了转变发展模式的新路子。经济增长方式开始向低投入、低能耗、高产出、高效益的方向转变。2004年，全市每平方公里土地产生1.75亿元的GDP和6000万元的税收，比1999年提高了1.4倍。万元GDP能耗0.55吨标准煤，万元GDP用水量47.2立方米，全口径财政收入占GDP的比重达34.6%，在全国处于领先水平。

　　自主创新能力增强，产业结构不断优化。专利申请和授权量逐年快速增长，发明专利的比重不断提高，成为全国第三大专利申请城市，PCT国际专利申请量已跃居全国第一。全市拥有自主知识产权的高新技术产品产值比重达56.7%。人才培养和引进工作得到加强，五年共引进各类人才42万，成为人才聚集的重要基地。华为、中兴等一批以自主创新为特征的民族高科技企业已初步成长为具有一定国际竞争力和全球影响力的企业。在平板显示、存储技术等一批电子信息产业高端领域初步形成发展优势。新材料、新能源和生物医药产业五年中产值翻了两番多。商贸、旅游、会展等服务业保持良好发展势头。成功举办首届深圳国际文化产业博览会，文化产业成为发展的新亮点。

　　推动经济、环境、社会协调发展的力度加大。大力实施“净畅宁工程”。加强生态城市建设，划定基本生态控制线，严把项目环保准入关，环保一票否决的项目年均超过1000项。全面启动水环境综合治理，加强机动车尾气污染防治，实现了工业污染源全面达标排放。先后获得国际“花园城市”、联合国环境保护“全球500佳”、“国家卫生城市”、“国家园林城市”、“全国绿化模范城市”等荣誉。从“梳理行动”到查处违法建筑和城中村改造，城市面貌发生了明显变化，违法抢建势头得到有效遏制。宝安、龙岗城市化工作取得重大进展，城市化转地工作全面启动。中心区基本建成。地铁一期如期通车，轨道交通二期项目获得国家批准。规划建设“一横八纵”干线路网，优化交通组织管理，公交优先战略取得初步成效，交通拥堵现象有所缓解。启动社会治安防控体系建设，强化流动人口、出租屋和娱乐场所管理，推行“网格化”布警，增加4500名警力，刑事案件高发势头初步得到控制，连续三次在全省社会治安综合治理考核中获得第一，被评为全国社会治安综合治理优秀地市。

　　（三）改革开放进一步深化，努力办出经济特区新特色。积极推动行政体制创新，通过两轮行政体制改革，行政审批事项减少37%，审批时限平均缩短了四成多。建立大企业便利直通车和政府部门与企业定期对话沟通制度，设立行政服务大厅。建设行政审批电子监察系统，对全市所有行政许可事项进行全过程监督。启动事业单位人事管理体制改革。全面推进综合财政预算体制改革，规范非税收入管理，强化财政资金使用的绩效审计和预算监督，进一步完善经营性土地使用权拍卖、政府工程招投标、产权交易市场和政府采购四项制度，清退违规借贷和逾期借款，不断创新预防腐败的工作机制，政府廉政建设成效明显。

　　国有企业改革和国有经济布局调整取得重要突破。一大批竞争性领域的劣势企业安全有序退出，国有资本主要集中在基础设施、公用事业和关系国计民生的重点领域，国有资产质量明显提高。国有企业的资产负债率大幅下降，财务风险基本解除，经营机制的转换有了新进展。对国有资产管理体制作了重大调整，国有资产的监管得到加强，重大决策失误、重大国有资产流失现象得到遏制。探索创新了行政事业性国有资产监管体系。积极支持个体私营等非公有制经济和中小企业发展，非公有制经济的活力和竞争力大大增强。全市私营企业年均增长30%左右。

　　落实国家宏观调控政策态度坚决，措施有力，成效明显。强化经济运行监测预警和调节，建立保障经济平稳运行的机制，及时加强发展中的薄弱环节，增强了驾驭市场经济的能力。积极应对全国性煤电油运供应紧张和水资源短缺问题，确保了城市正常的生活生产需要。全面加强知识产权保护，大力整顿和规范市场经济秩序。建立起比较完善的土地与房地产市场调控体系，有效遏制了投机炒作行为，保证了房地产市场的健康稳定。投融资体制改革、行业协会民间化改革、社会诚信体系建设、要素市场建设等工作取得积极进展。社会保障制度、医疗卫生体制等各方面的改革取得了新成果。

　　大力实施科技兴贸，努力开拓新兴市场。完善口岸通关服务，加快发展保税区、出口加工区，成立市WTO事务中心，积极应对贸易风险和摩擦。五年累计实际利用外商直接投资137亿美元。世界500强企业来深投资已逾百家。抓住入世和CEPA实施的有利时机，服务贸易领域对外开放步伐加快。

　　加强与香港多层次、多领域的合作，在粤港合作的框架下，建立深港政府间重大事项协商沟通机制，口岸和跨界基础设施合作不断加强，经贸、科技、教育、金融、旅游等合作不断深化。密切与珠三角各城市、泛珠三角各地区的合作，积极参与西部大开发、东北老工业基地振兴，加大了对口扶持力度。

　　（四）坚持以人为本，民主法制、社会事业和精神文明建设实现新发展。自觉接受人大的法律监督、工作监督和人民政协、民主党派的民主监督，做到重大决策事项事先征求人大、政协的意见。支持鼓励媒体的舆论监督，政府工作的透明度和市民对社会公共事务的参与度不断提高。五年来，市政府系统共办理人大议案7件、办复建议2193件、政协提案2677件，提请市人大审议法规议案26项，制定、修改和废止了一批政府规章。推行政府机构和行为的九个法定化，实行依法行政责任制和行政综合执法，严肃查处行政不作为和“乱作为”，政府依法行政意识增强，行政执法水平提高，行政诉讼败诉率逐年下降。

　　大力实施“文化立市”战略，积极推进文化体制改革，文化事业更加繁荣，群众性文体设施和网络不断完善，各类文体活动蓬勃开展。广播影视、新闻出版事业发展迅速，卫星电视频道开播，文艺精品不断涌现。基础教育初步实现均衡化和优质化，六个区全部建成省教育强区，我市成为全省第一个教育强市，高等教育发展加快。群众体育蓬勃发展，竞技体育取得突破，在两届奥运会上为国家赢得一金一铜。

　　成功抗击“非典”和禽流感疫情，积极推进公共卫生体系建设，完善疾病防控体系。组建了市应急指挥中心，形成了行之有效、协调有力的公共危机处置机制。认真实施食品放心工程，食品安全管理得到加强。高度重视抓好安全生产，五年未发生特大安全生产事故。畅通信访渠道，健全各级领导接访日制度，积极化解集体访及各类重点社会矛盾。不断完善就业服务体系，五年共帮助7万多人次实现再就业，城镇居民登记失业率控制在2.6%以内。制定并严格执行《员工工资支付条例》，社会保障体系日趋完善，覆盖面不断扩大，宝安、龙岗两区城市化人员全部纳入社保体系，劳务工社会保障不断加强。大力实施固本强基工程，基层组织和社区建设得到加强。认真做好困难群众的帮扶工作，“同富裕工程”取得成效。整合全市档案资源，档案工作信息化水平和服务水平走在全国前列。计划生育工作成绩显著。统计、外事、侨务、港澳台务、民族宗教、民防、气象等各项工作都取得新的成绩。

　　积极创建“全国文明城市”。在广大干部群众中深入进行邓小平理论和“三个代表”重要思想学习教育。加强公民道德建设，推行市民行为道德规范，通过开展“深圳关爱行动”、“深圳读书月”等系列活动，全面提升市民的文明素质，深圳精神的内涵更加丰富。连续三次被评为“全国双拥模范城”，连续五次评为省“双拥模范城”，国防教育、国防后备力量建设得到加强。

　　各位代表！过去五年，我们所取得的每一项成绩，每一点进步，都是党中央、国务院、省委、省政府和市委坚强领导的结果，都凝聚了全市人民的辛勤劳动和聪明才智。在此，我代表本届政府，向奋斗在各条战线的全市广大干部群众及外地来深建设者，向给予我们支持的人大代表、政协委员、各民主党派、工商联和社会各界人士，向中央和各省区市驻深单位，驻深解放军、武警官兵，向所有关心和支持深圳发展的港澳台同胞、海外侨胞和国际友人表示崇高的敬意和衷心的感谢！

　　我们也清醒地看到，前进的道路上面临的困难和问题还很多：经济社会发展的体制机制性障碍比较多；产业结构在国际分工体系中仍处于较低层次，发展的资源、环境、生态成本比较高，水、电、土地等资源约束趋紧；城市人口增长过快；河流污染比较严重，空气质量下降，治理效果尚不明显；社会治安问题比较突出，刑事案件发案仍在高位徘徊；查处违法建筑、违法用地和城中村改造的任务比较艰巨；群体性事件呈上升趋势，妥善处理和化解社会矛盾的机制尚不健全；政府社会管理和公共服务水平还不够高，执行力还不够强，一些部门之间职责不清、推诿扯皮的现象依然存在。

　　我们要增强忧患意识和责任感，坚定信心，知难而进，认真采取措施解决以上问题，不断实现新的发展，绝不辜负全市人民的重托和期望。

　　二、今后五年的目标任务

　　今后五年是深圳充满机遇又面临重大挑战的五年，是探索新的发展模式并实实在在取得成效的关键五年。

　　刚刚闭幕的市第四次党代会提出了深圳今后五年工作的总体要求：高举邓小平理论和“三个代表”重要思想伟大旗帜，全面贯彻党的十六大精神，以科学发展观统领经济社会发展全局，以改革创新为动力，以全面加强党的建设提高执政能力为保证，努力建设和谐深圳效益深圳，力争在2010年基本实现社会主义现代化，经济总量翻一番，人均GDP达到12000美元左右，社会事业全面进步，人民生活水平和质量明显提高，城市竞争力显著增强，为建设国际化城市奠定坚实基础。

　　按照和谐深圳效益深圳的要求，我们不仅要实现产出水平、产出能力的提升，还要实现单位产出的资源、能源消耗水平下降；不仅要注重经济效益，还要注重生态效益；不仅要促进社会进步，还要促进人的全面发展，把深圳建设成为经济繁荣、资源节约、环境优美、功能完备、文明法治、安定和谐、最适合人居和创业的城市，让我们的市民为自己的城市感到由衷的自豪。

　　我们要按照党代会的总体要求，依据未来五年的目标和任务，制定可操作、可考核的政策措施和工作步骤，以改革创新为动力，以提高执行力为重点，加大力度，扎扎实实抓好落实。在工作指导和安排上，突出三个方面：第一，要建立一个面向未来、创新活跃、具有持续增长动力和全球竞争力的产业体系；第二，要建设一个文明法治、和谐有序，崇尚创新、宽容失败的社会发展环境；第三，要建立起责任明晰、执行力强、依法行政、廉洁高效的服务型政府。

　　（一）以转变增长方式，提高效益为核心做好各项经济工作。

　　未来五年，建设效益深圳的关键就是要增强产业竞争力，做大做强高新技术、物流、金融、文化四大支柱产业，努力提升我市制造业在国际分工中的地位，努力提升服务业发展水平。

　　强化制度创新和技术创新，实施适度重型化战略是适应我国、我市经济发展阶段的重大战略举措，是建设效益深圳的重要内容。未来五年，要加快高新技术产业向产业链、价值链高端升级，加快从加工装配向设计制造转变，加快发展电子信息、先进装备制造、汽车及零配件、精细化工、生物医药及新能源、新材料产业，加快以高新技术和先进适用技术改造有市场潜力的传统优势产业，提高装备水平、技术水平和工艺水平，走出一条具有深圳特色的新型工业化道路。

　　要切实加快发展物流、金融、文化产业以及商贸、旅游、会展、中介等现代服务业，在更广的范围和更高的层次拓展服务业的发展空间，增强城市的综合服务功能和对外辐射能力，促进二、三产业的良性互动。

　　为此，要做好以下几个方面的工作：

　　第一，制定切实有效的产业发展政策。既要优化产业结构，提升产业效益，又要保持产业发展的消长平衡，要从单位面积投资强度、产出水平及能源资源消耗标准等方面，调整不同产业类型的准入门槛，保证新增产业的高效益，促进产业存量的结构调整和优胜劣汰，让高消耗、高污染、低效益产业为优势产业腾出发展空间。未来五年，要实现全市万元GDP的建设用地年均下降10%，工业全员劳动生产率年均增长5%，万元GDP的能耗、水耗逐步下降。要按照“南软北硬、南高北重、东西两翼港口旅游”的空间布局战略，调控城市产业布局，使特区外单位土地产出效益达到目前特区内的平均水平，大工业区、光明高新区、宝龙工业区等主要工业园区的单位土地产出效益达到目前市高新区的水平。

　　第二，进一步完善区域创新体系。积极探索具有深圳特色的自主创新之路，建设科技创新的特区，成为国家自主创新战略的试验田。加强重点实验室、工程中心、科技企业孵化器、技术标准检测等公共技术平台和企业博士后流动站建设，积极组织重大关键技术联合攻关，努力在重点领域形成产业创新高地。完善技术产权交易市场体系，支持创投业的发展，积极促进国内外研发成果在我市实现产业化。大力实施技术标准战略和名牌带动战略，切实把我市的专利优势转化为产品优势、企业优势和产业优势，培育自己更多的名牌产品和有国际竞争力、国际影响力的一流企业。加强知识产权保护，全力推进“国家知识产权试点城市”建设。激发和保护好企业家群体的创新热情，发挥企业家在科技创新中的作用。积极培育创新文化，大力发展动漫、创意设计等文化产业，促进文化与市场、文化与科技的对接，努力把高交会和文博会办成国际知名展会。

　　金融创新是构建区域创新体系的重要环节。要大力推动银行、证券、保险业在业务、技术、组织等方面加强合作，积极支持金融创新，形成“大金融”的发展格局，巩固和加强区域性金融中心城市的地位。推进多层次资本市场体系建设，支持深交所中小企业板和债券市场不断发展壮大。加强深港金融合作，提高深圳金融业的国际化水平。通过引进战略投资者、完善法人治理结构、提高资本充足率和资产质量等，积极推进市属金融机构的改革和整合。加快金融聚集区的规划建设，继续做好政策支持、服务和协调工作，完善企业征信和个人征信体系，营造良好的金融发展环境。积极配合各监管机构建立健全金融系统风险预警机制，协助金融机构完善内部控制体系和风险防范体系，认真做好金融稳定工作，努力建设金融运行安全区。

　　第三，提高开放型经济的质量和效益。努力实现外贸出口从低附加值、简单加工为主向高附加值、自有品牌为主转变。完善外贸争端应对和服务机制，建立产业损害预警机制，维护产业安全。全面推进“大通关”工作，完善口岸通关服务。优化利用外资结构，重点引进有利于完善产业链的关键项目，不断扩大园区招商比重，吸引跨国公司把地区总部、高附加值的制造环节、研发中心、采购中心和服务外包基地设在深圳。鼓励企业“走出去”，利用全球资源扩大发展空间，拓展国际市场。全面推进与香港的科技、教育、服务贸易合作，提升深港经济合作与产业分工水平。落实《深圳市融入泛珠三角区域合作实施方案》，加强与珠三角、泛珠三角的交通、资源、能源、环保等合作。

　　第四，大力增强城市功能和辐射力。未来五年，要加快推进京广深、杭福深高速客运专线深圳段建设，加快新深圳站、深圳集装箱中心站的建设，以“两线两站”为标志，把深圳建成全国重要的铁路枢纽城市。这是从根本上改变我市在国家交通布局中地位的重大战略机遇，我们必须紧紧抓住，丝毫不能放松。积极推进港口重大项目建设，实现深圳从国际集装箱大港到强港的跨越。加快推进深圳机场飞行区扩建工程，进一步提升深圳机场航空货运中心地位。完善中心区功能，规划培育和改造提升一批重点商圈，加快物流、商贸、旅游等基础设施建设，提高服务业的发展水平和辐射能力。全面推进覆盖全市的轨道交通建设，构建网络化的轨道交通体系。抓好“一横八纵”城市干线路网建设，滚动推进“七横十三纵”路网远期规划的实施，使特区内外的交通联系更加顺畅便捷，推动城市一体化发展。积极推动沿江高速、广深铁路四线等省重点项目建设。完成西部通道、皇岗和文锦渡口岸改造等跨境基础设施建设。加大引水工程、供水网络和电力基础设施建设力度，完善能源和水资源保障体系。继续完善政府投资项目代建制，积极扩大投资项目储备库，加强重大项目的前期研究，实施重大项目责任制，促进固定资产投资持续稳定增长。

　　第五，加大科技、教育、环保投入力度。完善科技投入体系，优化科技资源的配置方式，充分发挥政府科技扶持资金的杠杆作用，不断提升科技创新与知识经济对经济增长的贡献。大力推进教育现代化，促进人的现代化和劳动生产率的提高。继续加强基础教育，突出素质教育，进一步办好深圳艺术学校等有特色的中专和技工学校，整顿、规范、支持民办教育。加快发展高等教育，把深圳大学办成教学与科研并重、若干专业在全国领先的综合型大学，继续保持深圳职业技术学院在全国职业教育中的优势地位，努力将深圳信息职业技术学院办成国家级示范性软件学院，继续以改革的精神办好大学城，尽快形成办学规模，使之成为培养高级人才和推动科技创新的基地。进一步加大市区两级政府环保投入的力度，发挥社会资本投资环保产业的积极性，促进环境基础设施建设与运营的市场化，使全社会环保投入占GDP的比重与我市经济社会发展水平更相适应。

　　第六，促进循环经济的发展和节约型城市的建设。要抓紧研究制定行业节能降耗标准和措施，推行节能审计，鼓励开展资源节约的研究开发和技术改造，大力推广节能降耗的新设备、新工艺和新技术，推动各类企业向资源节约和清洁生产转型。按照循环经济的理念和要求把若干产业园区建成循环经济示范区。政府部门要成为建设节约型社会的模范，充分运用立法、价格等手段促进资源的节约和循环使用。大力推动海水利用和中水回用，积极创建节水型城市。

　　（二）以增进和谐为核心全面推进社会发展。

　　建设和谐深圳，就是要实现社会主义物质文明、政治文明、精神文明的协调发展，建设民主法治、公平正义、诚信友爱、充满活力、安定有序、人与自然和谐相处的社会，努力实现权利公平、机会公平、分配公平，保证公民平等享有法律规定的各项权利，共享发展成果。

　　为此，必须扎实推进以下工作：

　　第一，积极推进经济社会环境协调发展。坚持不懈实施“净畅宁工程”。“净”的问题最突出的是河流污染问题。过去五年，市政府在治理河流污染方面投入了大量人力物力，取得了初步效果，但距人民群众的期望还有明显差距。河流污染治理是一项系统工程，国内外河流治理短则十几年，长则上百年。深圳河流普遍径流量小，生态补水和纳污能力很差，治理的难度非常大。经过努力，已经形成了系统全面的治理方案，在完成专家论证后，年内将启动实施。治河的同时，要抓紧做好排污管网建设和错接乱排纠治工作，切实加强饮用水源保护。加大空气污染防治力度，重点治理机动车尾气污染，推广环保车型，加快电厂脱硫和“油改气”工作进度。要加强与周边各市合作，共同治理珠三角空气污染。做好城市卫生保洁、垃圾处理等工作，建立市容环境管理长效机制。加快生态城市建设步伐，加强基本生态控制线管理，全面实施规划环境影响评价制度。继续推进生态示范区建设，全面完成裸露山体和采石取土场的整治、复绿工作，新建一批市政公园、森林公园、郊野公园、社区公园和生态风景林。继续推进海洋生态环境和海岸线自然景观保护，严格控制东部海岸的开发强度，实现生态环境质量明显好转。

　　要实行最严格的土地管理制度，加强土地资源的保护和储备。推进旧城区的二次开发和改造提升，妥善处理历史遗留违法建筑和违法用地问题，积极做好违法建筑查处和城中村改造工作，使特区内城中村，特区外城市干道两侧、重要功能区内及各街道中心地段城中村的环境得到普遍改善，违法建筑得到全面整治。

　　实现城市交通的“畅”，要在抓好交通整体规划和重大基础设施建设的基础上，全面实施公交优先战略，完善轨道交通体系，推动快速大容量公共交通的发展，加大公交企业的重组，提高公交企业的规模经营水平和服务质量。加强交通组织，完善公交线路和行人过街系统，推进特区内外公交一体化。通过公共政策和市场机制的引导，改变居民出行方式，使公共交通分担率提高到50%以上。要严格交通秩序管理，加强交通联合整治，减少违章率，使交通拥堵情况得到显著改善，市民出行更加从容便捷。

　　要多管齐下解决“宁”的问题。进一步处理好社会治安管理与社会管理、城市管理的关系，全面落实社会治安综合治理责任制。积极推进公安改革工作，建立现代警务机制。加强公安正规化建设，加大装备投入，积极创建科技强警示范市。继续充实警力，精简机关，警力下沉，警务前移，全面提高警队的实战能力。扎实推进社区警务建设，改进暂住人口和出租屋管理，完善社会治安防控体系建设，加大对各类刑事犯罪特别是严重暴力性犯罪的打击力度，五年内实现社会治安明显好转。进一步完善安全生产责任制，强化安全监督管理执法，建立现代安全教育基地，预防和减少安全事故发生，切实保障人民群众生命财产安全。

　　第二，积极推进民主法制建设。和谐社会是法治社会。只有每位社会成员都遵纪守法，社会才能安定有序、文明和谐。要充分发挥法治在促进和保障社会和谐方面的重要作用，大力推进依法治市工作。进一步增强全社会的法律意识，无论是政府还是市民，都要依法办事并承担相应的法律责任。积极推进立法工作和责任体系建设，稳妥实施司法体制改革，严格执法，公正执法，努力维护社会公平正义。加强普法宣传教育，弘扬法治精神，引导市民自觉遵循社会规则、规章制度和公共秩序，以合法的方式表达诉求、用法律手段维护自身权益，形成人人知法守法的良好氛围。

　　健全政府与市民的沟通机制。完善新闻发言人和政府规范性文件公告制度，健全政府重大决策公示、听证制度，拓宽市民对公共事务的参与渠道。政府要主动与市民沟通，提高政府工作的透明度，善于运用宣传、解释和引导等方法化解矛盾，争取市民对政府工作的理解支持，这对于保障依法行政，增进社会和谐有着非常重要的意义。

　　政府要自觉坚持向人大及其常委会报告工作制度，对人大负责，接受人大的法律监督和工作监督。充分尊重和发挥人民政协、各民主党派、人民团体在政治协商、民主监督和参政议政中的作用，认真做好人大议案、建议和政协提案、建议案的办理，充分发挥新闻舆论和人民群众的监督作用。加强基层民主政治建设，积极推进居委会直接选举，提高居民自治能力，增强社区自治功能。

　　第三，切实关注社会民生。不断改善市民生活，继续保持居民收入水平全国领先，让全市人民实实在在地分享到经济发展的成果，得到更多的实惠。实施积极的就业和再就业政策，健全公共就业服务和职业培训体系，完善就业援助和创业资助制度，发挥创业促进就业的作用，使城镇居民登记失业率控制在3%以内。规范企业用工行为，打击劳务诈骗和欠薪等违法行为，切实维护劳动者的合法权益。研究解决社会成员之间收入差距过大的问题，规范分配秩序，加强对垄断行业收入分配的调控和监管。

　　进一步完善社会保障体系，扩大社会保险覆盖面。完善事业单位职员养老保险制度，探索建立完善劳务工的社会保障体系。规范社会保障基金管理，加强基金使用的审计、稽核和监察。全面建立疾病预防控制体系和突发公共卫生事件医疗救治体系，完善医疗基础设施建设，大力发展医疗中心和社区卫生服务，使市民享受到与城市发展水平相适应的优质医疗服务。加强食品药品监管，切实保障食品药品安全。积极推动与居民承受能力相适应的中低价经济适用房和廉租房建设。继续实施“同富裕工程”，推动我市欠发达地区加快发展。完善社会救助体系，积极支持老龄工作、残疾人事业和社会福利事业，大力发展慈善事业。

　　加强和改进新形势下的信访工作，认真抓好《信访条例》的贯彻实施，及时合理解决群众反映的问题，积极妥善处理群体性事件。健全社会矛盾纠纷排查调处机制，充分发挥人民调解的作用，做好法律援助工作。完善社会预警体系和社会动员机制，提高保障公共安全和处置突发事件的能力。

　　第四，加强精神文明和各项社会事业建设。大力弘扬深圳精神，实施公民道德建设纲要，重视和加强未成年人的思想道德教育。深入开展“深圳关爱行动”，积极发展义工事业，在全社会倡导关爱家园、团结互助、扶贫济困的良好风尚，形成平等友爱、融洽和谐的社会氛围，增强社会凝聚力和向心力，力争成为首批“全国文明城市”。

　　继续实施“文化立市”战略，弘扬先进文化。加强文化基础设施建设和文化遗产保护，积极吸引文化人才，繁荣哲学社会科学和文艺精品创作，发展广播影视、新闻出版、文艺展演等文化事业，加强“两城一都”和动漫产业基地建设。办好文化节庆和群众性文化活动，丰富社区文化。完善公共体育设施规划建设，开展全民健身活动，提高市民身体素质，办好重大体育赛事。做好妇女儿童工作，维护妇女儿童权益。稳定低生育水平，提高出生人口素质，重点加强流动人口计划生育管理和服务。增强大局意识，做好对口扶持工作。认真开展国防安全教育，加强国防动员和人民防空，积极开展双拥工作。支持工、青、妇等人民团体发挥好桥梁和纽带作用。做好统计、外事、侨务、港澳台务、民族宗教、边防、保密、档案、气象等各项工作。

　　（三）大力推进改革创新，努力突破建设和谐深圳效益深圳的体制机制性障碍。

　　改革创新是深圳的根，深圳的魂。我们要树立和强化特别能改革、特别能创新、特别能开放的意识，不断开创改革事业的新境界，以特别之为，立特区之位。未来五年，要立足于提高经济社会效益，立足于提升城市综合竞争力，立足于增进全市最广大群众的根本利益，重点推进经济和社会领域的八大改革。

　　一是加强和规范市场体系建设。积极发展资本市场、技术市场和产权交易市场，规范人才市场。继续深化土地管理制度改革，推进土地资源的市场化配置。加快垄断性资源和垄断性服务行业的市场化改革，政府要加强管理，严格控制价格水平，还要适当引入竞争机制，提高市场配置资源的程度和效率。积极推进行业协会的民间化改革，培育、规范和发展市场中介组织。深入整顿和规范市场秩序，严厉打击侵犯知识产权、制假售假、商业欺诈、偷逃骗税、走私贩私等不法行为。加强社会诚信体系建设，积极探索建立失信惩戒机制。

　　二是积极推进财税体制改革。建立完善的公共财政体制，是建设服务型政府的制度保证。加快推进财政预算改革，提高部门预算的水平和覆盖面，开展财政支出绩效评价体系改革试点，降低政府运行成本，提高财政资金的使用效益。按照财权事权统一的原则，进一步完善市区财政体系，明确两级政府的支出责任。深化非税收入改革，逐步扩大预算管理的范围，研究建立科学的政府收支分类体系。完善政府采购招投标制度，加强对采购活动的监管。落实国家税制改革措施，深化地方税收征管改革。

　　三是深化国有资产和国有企业改革。按照有进有退、有所为有所不为的原则，继续加快国有经济布局的调整。全面推进国有企业产权主体多元化，加快经营机制转换。大力提高企业核心竞争力，在公用事业、基础产业等领域做大做强一批产业集团，提高国有经济的影响力。进一步完善国有资产管理体制，加强对国有资产的监督，降低经营管理成本，全面提高国有企业的经济效益和社会效益。大胆探索和创新行政性国有资产的监管体系，加强对行政性国有资产的监督管理。按照政企分开、事企分开的原则全面剥离行政事业单位的经营性资产。积极推进集体股份公司改革。

　　积极鼓励、支持和引导非公有制经济发展，依法保护非公有制企业的合法权益，消除制约非公有制经济发展的体制性、政策性障碍，创造平等竞争的法治环境、政策环境和市场环境。改进政府对非公有制经济的服务和监管。非公有制企业要提高自身素质，遵守法律法规和安全、环保规定，保障职工合法权益。

　　四是积极推动投融资体制改革。引导、鼓励社会资本进入基础设施、公用事业以及法律法规未禁止的其他行业和领域。完善中小企业融资体系，拓宽非公有制企业融资渠道。进一步落实企业投资自主权，逐步放松对投资、技改的审批和审核。改革政府投资管理体制，完善投资主体和项目责任制，从根本上改变投资决策无人问责的状况。积极探索利用资本市场和各种金融工具创新政府融资方式，提高资金运作能力，发挥财政投入的放大效应，实现城市建设资金滚动发展，为金融市场注入新的活力。

　　五是推进城市管理体制创新。以国际化城市的标准提高管理水平，做到科学、严格、精细、长效，实现城市发展由建设为主向建管并重转变。适应城市化需要，统一特区内外规划建设管理标准，加快特区外向现代城市管理体制转变，使特区外城市面貌得到根本性改善。按照责权统一、管事与管人相一致的原则，合理调整市、区、街行政管理职能和权限，强化市政府对空间资源的集中统一管理，加强基层政府的社会管理职能，实现社会管理重心下移。坚持规划的刚性和法定性原则，充分发挥城市规划的先导、主导和统筹作用，建立规划实施的长效保障机制和部门协同机制，确保规划的实施效果。加快特区外法定图则编制工作，实现对规划建设区的全覆盖。

　　六是大力推动社会管理体制改革。加大社区建设投入，整合社区资源，全面设立社区工作站，健全社区群众自治组织，完善社区管理服务。坚持培育、发展和监管并重的方针，规划发展自律性、互助性和公益性的民间组织，充分发挥其提供服务、反映诉求和规范行为的作用，形成社会管理的合力。

　　七是深化人口管理体制改革。尽快推出加强人口管理工作的相关政策，创新人口管理手段，建立人口长效管理机制。根据城市发展需要和承载能力，合理控制人口总量，调整优化人口结构和空间布局。发挥产业政策对人口的调控作用，降低初级劳务工比重。大力实施人才强市战略，积极引进各类高素质人才，及时将符合城市发展需要的各类人才转为户籍人口，适当扩大户籍人口规模和比例，增强城市社会结构的稳定性。

　　八是全面推行各项社会事业改革。加大财政对社会公共事业的投入力度，加快公益性社会事业发展，通过二次分配促进社会公平。积极推动教育综合改革，扩大教育资源，提高教育水平，合理规范教育收费。深化医疗卫生体制改革，进一步规范药品政府采购制度，规范医疗服务收费，提高医疗水平。进一步深化文化体制改革，构建公共文化服务体系，积极培育文化市场主体，促进文化事业的全面繁荣和文化产业的快速发展。加快推进事业单位人事管理制度改革，增强事业单位活力，提高服务质量。

　　三、以提高行政执行力为重点，全面加强政府自身建设。

　　二十一世纪是经济全球化的世纪，任何一个国家和地区都要面对全球竞争的挑战。党中央、国务院就是从这样一个战略高度提出了加强执政能力建设，提高政府行政执行力，加快政府自身改革的总体要求。对于深圳而言，建设和谐深圳效益深圳，探索新的发展模式，更要加强政府自身建设。

　　一是深化政府机构改革。现代政府的一个特征就是职责清、任务明、交叉少、可考核、执行能力强。要进一步理顺部门之间的职能分工，重点解决职能交叉重叠、责任不清、事权分离的问题，明确每个部门、每个公务员所肩负的职责和责任，实现政府职能、机构、编制的科学化、规范化、法定化，使政府成为可考核的政府，公务员成为可考核的公务员。优化部门内部的人员配置，把更多力量投入到执法一线。进一步转变政府职能，凡是市场机制和社会自治能解决的，政府都要坚决退出，把主要精力用于加强社会管理和公共服务。建立行政审批事项审查和评价机制，把阶段性的审批制度改革变为经常性改革，该放的要放，该管的要管好。

　　二是严格依法行政。依法行政是提高行政执行力的关键环节。要认真落实《全面推进依法行政实施纲要》，率先建设法治政府。严格按照法定权限和程序行使权力，履行职责，接受监督。实行行政执法责任制，加强部门间的执法协调，克服多头执法、执法不公、执法不力的现象。既要认真做好行政复议，重视行政诉讼的应诉工作，及时纠正执法偏差，又要树立执法权威，做到坚决执法、严格执法、敢于执法，提高政府公信力。对于违法违规行为，不能坐视不理，更不能无原则地纵容退让。

　　三是加强行政效能监督和责任追究。大力推进政务公开，让政府的工作时刻处于人民群众和社会舆论的监督之下。完善行政效能监察体系建设，把政府行为的全过程纳入行政效能监察范围之内，进一步扩大绩效审计范围，推进绩效审计的制度化、规范化，充分发挥绩效审计在提高政府效率、规范行政行为中的作用，使行政监督贯穿于行政决策、行政执行的每一个环节。建立和完善行政责任追究制，对行政不作为，有令不行，推诿扯皮，影响行政执行力的行为严格追究责任，确保政令畅通。

　　四是大力加强公务员队伍和政风建设。认真贯彻《公务员法》，加强公务员队伍的培训、教育和管理，提高公务员队伍的整体素质和履职能力，吸引更多优秀人才进入公务员队伍。坚持求真务实，牢固树立正确的政绩观，坚决反对形式主义、铺张浪费、盲目攀比、急功近利等不良作风，节约行政成本，改进会风和文风，重实干、办实事、求实效。大兴调查研究之风，加快建设学习型政府，不断推动管理服务创新，提高落实科学发展观的能力和行政服务水平。大力加强廉政建设和反腐败斗争，严厉查处违法违纪案件，坚决纠正损害人民群众利益的不正之风。以保持共产党员先进性教育活动促进政府各项工作。

　　五是进一步提高政府战略决策水平。深圳经济社会持续快速发展，始终保持着旺盛的活力，一个重要的原因是历届市委市政府抓住了一系列战略机遇，避开了一系列风险，能够做到这一点，关键靠两条，一是从全国改革开放的战略高度出发，以超前的眼光想大事、谋大局；二是在实践中初步形成了防止战略决策失误的民主科学决策机制。当前，面对激烈的国际国内竞争，我们必须以特别能改革、特别能创新、特别能开放的精神，认真探索、紧紧把握关乎深圳长期发展的战略机遇、战略关键、战略要点。进一步完善民主科学决策机制，整合培育决策咨询研究队伍，发挥专家和研究机构在战略决策中的作用；进一步完善政府内部决策规则和行政决策程序；规范与完善重大决策事先征求人大、政协意见的工作制度和程序；探索建立决策实施效果的评价机制，不断提升战略决策水平。

　　四、对2005年政府重点工作的建议

　　2005年，是全面完成“十五”任务、为“十一五”发展打好基础的关键一年，也是建设和谐深圳效益深圳的起步之年。按照市委三届十一次全会和第四次党代会的总体要求，对今年政府重点工作提出以下建议，要实现万元GDP建设用地下降10%、万元GDP能耗和水耗有所下降、空气质量和河流水质有所改善，在建设和谐深圳效益深圳的道路上迈出实实在在的步伐。同时，建议今年经济社会发展的预期目标是：本市生产总值增长13%，全口径财政收入占本市生产总值比重达到35%左右，城镇登记失业率控制在3%以内，居民消费价格指数102%。

　　今年前五个月全市正朝着上述目标稳步发展，接下来要重点抓好以下十项工作：

　　一是认真做好“十一五”规划编制工作，积极争取把深圳发展的重大基础设施建设项目纳入国家“十一五”规划。加强空间政策的研究和制定，抓紧编制《深圳市近期建设规划（2006—2010）》，科学统筹城市建设的空间布局。制定和谐深圳效益深圳的指标体系，建立健全相关政策法规。

　　二是加快推进产业结构的优化升级。强化技术创新，提升自主知识产权水平。力争在产业结构适度重型化和电子信息产业的一些关键领域继续取得突破，做好精细化工产业园区、国家半导体照明产业化基地和生物医药产业化基地的规划建设。办好第七届高交会，加快华南技术产权交易中心建设，促进保税物流园区和保税物流中心发展，推进金融中心区建设，出台加快文化产业发展的配套政策，做好第二届文博会的筹备工作。支持传统优势产业发展，提高竞争力。

　　三是加强对改革的领导、统筹和协调，各级各部门主要领导要亲自抓改革，建立改革工作责任制，形成推动全市改革的有效协调机制和合力。重点在加强政府执行力、完善市场经济体制、加快事业单位改革、加强行政事业单位国有资产监管、强化社会管理等方面取得突破。

　　四是要在转变外贸增长方式，提高利用外资质量上取得实效。加快建立健全WTO公共服务平台，进出口预警系统和法律援助体系，帮助企业应对各种贸易摩擦。完善招商引资责任制，提高招商引资水平，着力引进产业层次高，经济、生态和社会效益比较好的项目，鼓励外商充分利用现有厂房和土地增资扩股。

　　五是高度重视协调解决好水、电、土地资源的瓶颈制约问题。加快推进东江水源二期、北线引水和梅林、笔架山、大工业区及大鹏半岛支线等工程建设，加大特区外饮用水管网建设，切实保证饮用水质量。统筹安排电源，加快电网建设，完善错峰用电方案和电力供应安全应急机制。研究建立能源储备制度。积极配合市人大加快循环经济立法，大力开展节能、节水活动。合理开发利用土地资源，提高土地集约利用水平。

　　六是认真做好城市化转地、违法建筑查处和城中村改造工作。能否按期完成转地工作，对于保证深圳未来的发展空间至关重要。对转地工作的难度要有充分的估计，要把转地工作摆在各级政府和各部门工作的突出位置，确保相关政策法规在转地工作中得到严格执行和及时、全面落实，确保转地工作如期完成。认真做好转地后土地资源的储备、控制和管理工作。严肃查处违法抢建和抢种行为，积极稳妥地推进城中村改造，确保完成年度工作任务。认真研究解决特区内城市化遗留问题。

　　七是加强督查督办，切实推进重大项目建设。启动轨道交通二期工程，1号、4号线延长段年内全面动工，2号、3号线前期工作加快推进。南坪快速路一期、清平路、盐排高速要全面建成通车，加快西部通道、福田交通枢纽换乘中心、福龙路、南光高速、深盐二通道等项目建设。进一步推进东部地区交通系统的规划建设。加快京广深和杭福深客运专线深圳段“两线两站”立项和前期工作。加快盐田、蛇口、赤湾、妈湾、大铲湾港口建设，力争开通深圳至昆明、长沙等地的“五定”班列。做好深圳机场飞行区扩建工程的前期工作。

　　八是大力实施珠江流域（深圳）水环境综合整治和治污保洁工程，启动深圳河湾水污染治理，实现新洲河、福田河、大沙河、盐田河四条流经市区的河流基本不黑不臭，积极推进布吉河、龙岗河、坪山河、观澜河等河流的整治。全面推进特区外污水收集处理设施建设。强化机动车尾气污染治理，加快电厂脱硫设施建设。做好“国家环保模范城”复查迎检工作和“全国文明城市”的创建工作。

　　九是切实搞好社会治安、维护稳定和安全生产工作，重点开展针对“涉黑”、严重暴力性、多发性侵财犯罪和重大经济犯罪的专项打击整治行动，推进出租屋旅业化、暂住人口信息化管理工作，完善路面治安防控网络，进一步遏制刑事犯罪，保障人民生命财产安全。积极推进各项社会事业发展，做好就业和再就业工作，完善社会救助体系和社区管理、自治、服务功能。

　　十是推进并完成一批教育、文化、卫生、体育重大基础设施建设。积极做好第二十六届世界大学生运动会申办工作。认真做好深圳经济特区成立25周年有关庆祝活动。

　　各位代表，建设和谐深圳效益深圳的大政方针已定，历史赋予我们的使命光荣而艰巨。我们要坚定信心，激扬志气，在全国各地加快发展的激烈竞争中挺立潮头，再创佳绩！让我们紧密团结在以胡锦涛同志为总书记的党中央周围，高举邓小平理论和“三个代表”重要思想伟大旗帜，全面贯彻党的十六大和十六届三中、四中全会精神，在省委省政府和市委的正确领导下，认真落实市第四次党代会确定的各项工作部署，开拓创新、锐意进取，建设深圳更加美好的明天！
